# Pinniped electroencephalography: Methodology and findings in California sea lions (*Zalophus californianus*)

**DOI:** 10.3389/fvets.2023.1040125

**Published:** 2023-03-31

**Authors:** D. Colette Williams, Martin Haulena, Sophie Dennison, Lynnette Waugh, Tracey Goldstein, Felicia Nutter, Bill Van Bonn, Vanessa Hoard, Kenneth D. Laxer, Paul S. Buckmaster, Frances M. D. Gulland, Barry Tharp

**Affiliations:** ^1^Vet EDX, Retired Veterinary Medical Teaching Hospital, University of California, Davis, Davis, CA, United States; ^2^The Vancouver Aquarium, Vancouver, BC, Canada; ^3^Imaging Solutions, PLLC, Oakton, VA, United States; ^4^UC Davis School of Veterinary Medicine, Davis, CA, United States; ^5^Zoological Pathology Program, University of Illinois at Urbana-Champaign, Brookfield, IL, United States; ^6^Department of Infectious Disease and Global Health, Cummings School of Veterinary Medicine, Tufts University, North Grafton, MA, United States; ^7^A. Watson Armour III Center for Animal Health and Welfare, Animal Care and Science Division, John G. Shedd Aquarium, Chicago, IL, United States; ^8^Department of Neurology, The Pacific Marine Mammal Center, Laguna Beach, CA, United States; ^9^Sutter Pacific Medical Foundation, San Francisco, CA, United States; ^10^Departments of Comparative Medicine and Neurology and Neurological Sciences, Stanford University, Stanford, CA, United States; ^11^The Marine Mammal Center, Sausalito, CA, United States; ^12^Wildlife Health Center, University of California, Davis, Davis, CA, United States; ^13^Emeritus, Department of Neurology, University of California Davis Medical Center, Sacramento, CA, United States

**Keywords:** seizures, epilepsy, domoic acid (DA), memory, spatial ability, EEG, hippocampus

## Abstract

This study was designed to identify abnormalities in the electroencephalograms (EEGs) recorded from stranded California sea lions (*Zalophus californianus*) with suspected *domoic acid (DA) toxicosis*. Recordings from animals presenting for non-neurological issues were also obtained to better understand the normal EEG (background activity and transient events) in this species, as, to date, studies have focused on examining natural sleep in pinnipeds. Most animals were sedated for electrode placement and EEG acquisition with some receiving antiepileptic medications or isoflurane during the procedure. A total of 103 recordings were read and scored from 0 (normal) to 3 (severely abnormal). Epileptiform discharges, consisting of spikes, sharp waves, slow waves, and/or spike waves, were present in all EEGs with scores of 1, 2, or 3. The distribution of these events over the scalp varied. While often generalized, others were lateralized over one hemisphere, bifrontal, bioccipital, and/or bitemporal, while some discharges were multifocal. Findings were different between sea lions and occasionally changed within the EEG on a given sea lion. No clinical seizures were observed during the recording but a few sea lions had findings consistent with electroencephalographic seizures. When available, supporting diagnostic results obtained from magnetic resonance imaging (MRI) and/or necropsy/histopathology were described, as well as the status of those sea lions that recovered and were released with satellite tags.

## Introduction

Credited with being the first to describe the human electroencephalogram (EEG) in 1929, Hans Berger's study (1) was expanded upon with the publication of petit mal (now called absence) epilepsy findings in 1935 (2). Epileptic events consisting of spike-wave discharges at 3 Hz and interictal spikes were described in this report by Gibbs, Davis, and Lennox (2). More studies have been conducted since then with EEG being considered a standard diagnostic tool in human medicine for a variety of disorders, especially epilepsy. This diagnostic technique remains comparatively rare in veterinary medicine with studies available at some academic institutions and large specialty practices but primarily limited to companion animals. Brain wave activity has been recorded from marine mammals in research settings. Several earlier publications describe the use of implanted electrodes to study natural sleep in multiple species of cetaceans (toothed whales and dolphins) (3–5) and pinnipeds (seals and sea lions) (6–9). Recent studies have employed non-invasive methods for recording EEG in the latter (10–12). Sleep background patterns were noted in only one hemisphere when marine mammals were submerged but were bilateral when resting at the surface or, in the case of pinnipeds, hauled out. Other than brief descriptions involving some of the sea lions presented here (13–16), detailed findings of clinical EEGs recorded from *Zalophus californianus* have not been reported.

Recurrent seizures in humans and sea lions have been associated with the consumption of seafood (fish or shellfish) that has ingested certain algal diatoms containing domoic acid (DA). In late 1987, 150 people in Canada ate blue mussels that contained *Pseudo-nitzschia multiseries* that had been collected from nearby Prince Edward Island River (17). Human patients (107 confirmed cases) presented with gastrointestinal symptoms within 24 h or exhibited at least one neurological symptom within 48 h of ingestion. The latter consisted of memory loss, confusion, disorientation, seizures, coma, or cranial nerve palsies. Four people died within 24 days of mussel consumption. Several of those people who were severely affected had anterograde amnesia, resulting in the disease being given the moniker, *amnesic shellfish poisoning*. Seizures were a common finding in severe cases but lessened in frequency over a 2-month period. An 84-year-old male patient went from being disoriented and sleepy the day following shellfish ingestion to comatose with complex partial status epilepticus by day 3 (18). On EEG, he was found to have diffuse background slowing and lateralized periodic discharges (PDs) on the left side and bitemporal independent epileptic events. He was discharged seizure-free 4.5 m later and had a normal EEG 8 m after exposure, although he continued to have severe memory impairment. A year after acute DA exposure, he developed complex partial seizures and was diagnosed with temporal lobe epilepsy. At that time, magnetic resonance imaging (MRI) demonstrated bilateral hippocampal atrophy. An autopsy 2 years later, following his death from pneumonia, revealed sclerosis of both hippocampi and the mesial temporal region.

First, observed in 1998, multiple marine mammal mortality events have been reported along the Western US coastline. California sea lions, long-beaked common dolphins (*Delphinus capensis*), short-beaked common dolphins (*Delphinus delphis*), bottlenose dolphins (*Tursiops truncatus*), gray whales (*Eschrichtius robustus*), and a single northern fur seal (*Callorhinus ursinus*) were all thought to have died after acute DA exposure (19, 20). Northern anchovies (*Engraulis mordax*) were considered the primary vector in the sea lion cases (21). More recently, a large number of sea lions have been stranded with health issues attributed to chronic exposure to DA. In addition to neurological manifestations (13), reproductive failure (22, 23) and a degenerative cardiomyopathy (24) have also been associated with the toxin. Affected sea lions, similar to humans with amnesic shellfish poisoning, have also been shown to have memory deficits (25). There is evidence that some cases may have resulted from exposure *in utero* or neonatally, as DA has been found in amniotic fluid and dam's milk (26). The odd behavior described in sooty shearwaters in 1961 (27), which was the inspiration for Hitchcock's movie, *The Birds*, is thought to have been associated with an earlier *Pseudo-nitzschia* plankton bloom (28, 29). Multiple mass die-offs of brown pelicans and Brandt's cormorants in Monterey Bay, California, in each of the following three decades were determined to be DA-related diseases after finding contaminated anchovies in the stomach contents of affected birds (29).

In this retrospective study, EEG was used in an attempt to identify sea lions with suspected neurological damage subsequent to *DA toxicosis*, a common cause of strandings in this species. Sea lions are exposed to DA by ingestion of fish that have consumed the marine diatom *Pseudo-nitzschia australis*.

## Materials and methods

Over a 7-year period (2004–2011), electroencephalographic studies (EEGs) were performed on 120 California sea lions that were rescued by The Marine Mammal Center following stranding along the California coast. Most animals were found between Sonoma County and San Luis Obispo County with the exception of one individual in the Sacramento delta.

### Sea lions

Animals were divided into two groups based on signs at presentation. The Neurologically Normal Group consisted of 15 sea lions. There were a total of 88 animals in the group designated as the Suspected DA Toxicosis Group. Notably, 12 sea lions, two that would have been in the neurologically normal group and ten with suspected DA toxicosis, received isoflurane at the beginning of their recordings, making interpretation difficult. These were excluded from the study. Another five, all DA suspects, had unreadable recordings (corrupted files). This brought the total number of sea lions evaluated down from 120 to 103.

*Neurologically Normal Group* presented with various clinical problems, including malnutrition, leptospirosis, trauma, entanglement, or abscess. Three had no clinical signs and the cause of stranding was unknown. Eight were restrands (they had been previously admitted and tagged before release). Signs associated with central nervous system dysfunction were not observed in any of these sea lions on admission. This group consisted of 15 animals. With one exception, all animals in this group were young males. They were classified as pups (MP), if less than a year (*N* = 5), yearlings (MY), if 1 to 2 years (*N* = 3), juveniles (MJ), between 2 and 4 years (*N* = 3), and subadults (MS), if 4 to 8 years (*N* = 3). The lone female (FP) in this group was a pup (less than 1 year).

*Suspected DA Toxicosis Group* presented with neurological signs including, unresponsiveness, seizures, abnormal behaviors (head weaving, scratching, and flipper biting), ataxia, and/or aggressiveness. Blindness was noted in several sea lions, either initially or after time spent in rehabilitation. There were 88 animals in this group: 15 males (1 MP, 4 MY, 8 MJ, and 2 MS), 72 females (1 FP, 10 yearlings [FY], 10 subadults [FS, between 2 and 5 years], 45 adults [FA, >5 years], and 6 unknown ages),^1^ and one unknown gender (not recorded). There were 21 restrands in this group.

Several animals, especially those in the latter part of the study, had courses of phenobarbital prior to performing the EEG study. A total of 14 sea lions had additional recordings, one to two repeats. Some involved different sedative/anesthesia combinations or courses of antiepileptic medication between the repeat and the original recording. Those sea lions in which isoflurane was administered at the onset were not scored.

### EEG recording

**Figure 1:**
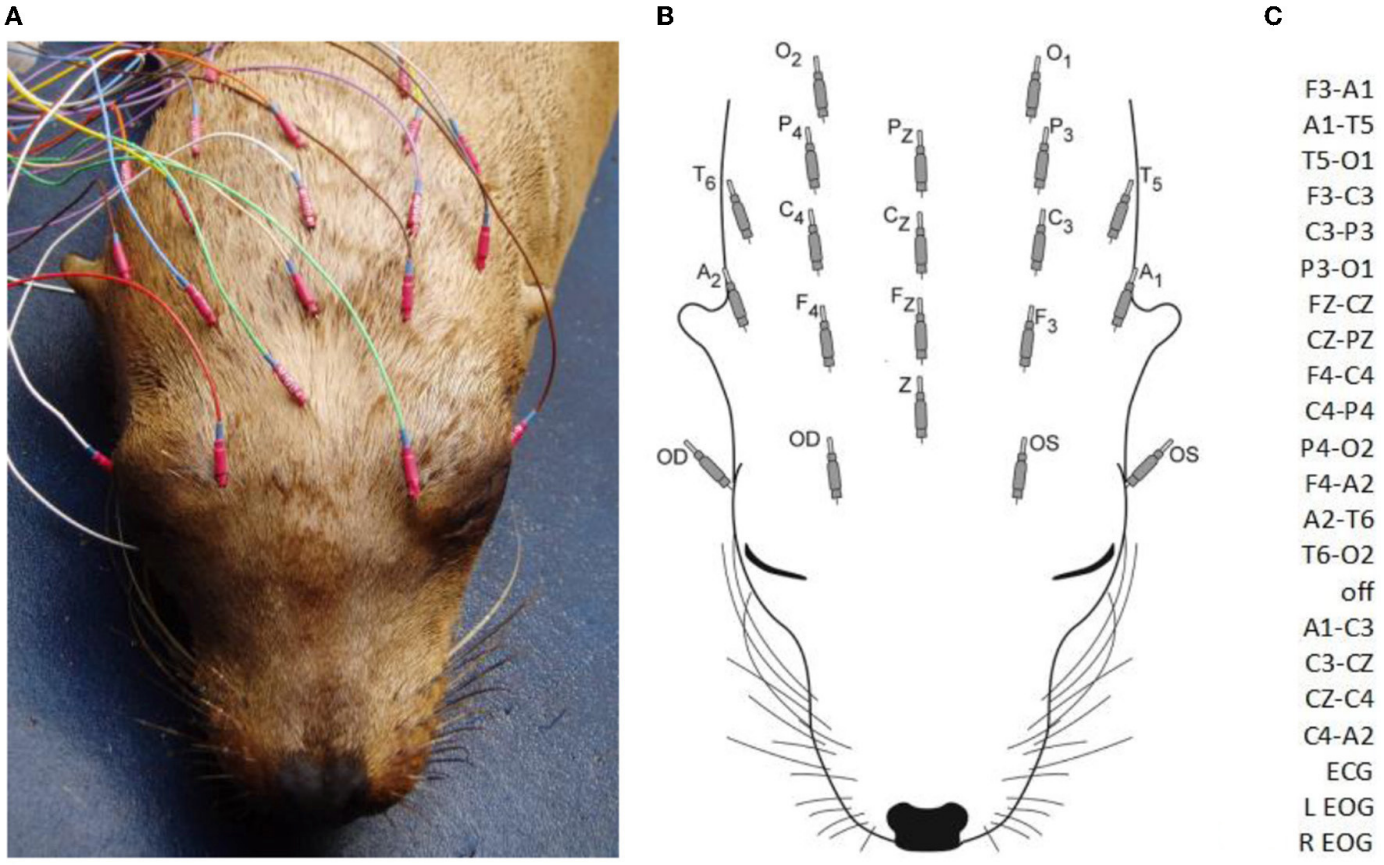
A sea lion with subcutaneous electrodes in place for EEG/EOG **(A)**. Only the hubs are visible (the 12 mm needles project rostrally). A diagram of electrodes with designated labels **(B)**. The modified double banana montage utilized **(C)**. F, frontal; C, central; P, parietal; O, occipital; T, temporal; A, aural; odd numbers, left side; even numbers, right side; z, midline; Z, ground; OS, left eye; OD, right eye (ECG not shown).

Sea lions were housed at the Marine Mammal Center in individual pens with freshwater or saltwater pools prior to 2009 and saltwater thereafter. They were transported to an enclosure at the surgical facility for EEG recordings at the discretion of the attending veterinarian. Except for two obtunded animals, sea lions received medetomidine at a dose of 0.07 mg/kg IM or dexmedetomidine at 0.35 mg/kg IM for sedation (to facilitate placement of electrodes). A total of 68 individuals were also administered 0.2 mg/kg butorphanol IM at the same time as the a_2_ agonist. After observing the effect of the sedatives, at which time the animals were resting quietly, 15 26-gauge platinum needle EEG electrodes (Grass Astromed) were inserted under the skin of the scalp. Due to similarities in skull shape, electrode placement was done based on that routinely used in mesocephalic dogs at the University of California, Davis Veterinary Medical Teaching Hospital. This consisted of three evenly spaced midline electrodes (F_Z_, C_Z_, and P_Z_), four each on the left (F_3_, C_3_, P_3_, O_1_) and right (F_4_, C_4_, P_4_, O_2_) with all frontal (F), central (C), and parietal (P) electrodes in the same transverse plane and two pairs of lateral electrodes (left and right), dorsal to the pinnae (A_1_ and A_2_), and caudal but in the same coronal plane (T_5_ and T_6_) as aural (A) electrodes (Figures 1A, B). A ground electrode (Z) was positioned over the frontal sinus at the midline. Additional electrodes were placed to record the electrooculogram (EOG) and the electrocardiogram (ECG), i.e., the former near the lateral and medial canthus of each eye (in most sea lions, they were not included in highly reactive animals) and the latter on the chest near each pectoral flipper. Electrodes were connected to a mini-input box (Nihon Kohden Inc.) which led to the amplifiers of the Neurofax 2100 digital EEG system (Nihon Kohden Inc.). Electrode impedances were checked and determined acceptable at 10 kΩ or less. The recording bandwidth was set with a time constant of 0.1 s and a high-frequency cutoff value of 70 Hz. Display sensitivities varied between 5 and 20 μV/mm (depending on the amplitude of the EEG signal from each sea lion), and the time base was 10 s/page. Short segments of a 50 μV/mm calibration signal and a biological calibration were recorded to insure proper amplifier function prior to recording. A double banana montage, based on that used in human electroencephalography, was utilized for both display and analysis of the recordings (Figure 1C). Some EEG segments were also reviewed with a referential montage. EEGs varied in length from 10 to 61 min, depending on patient tolerance for the procedure and time constraints. Five animals received midazolam IM and three received lorazepam IM during the EEG recording. Atipamezole at 0.25 mg/kg IM was administered at the end of the procedure to reverse the effects of the a_2_ agonists (medetomidine or dexmedetomidine) in 11 sea lions. When additional diagnostic procedures were required, animals were supplemented with isoflurane gas anesthesia with oxygen *via* face mask while recording continued (*N* = 22).

### EEG scoring

Most recordings were interpreted independently by two electroencephalographers (DCW, with a background in veterinary EEG, and BT, an expert in human EEG), while the latter recordings, sea lions numbered 9008 and above, were reviewed together. Only those sections following a_2_ agonist administration (with or without butorphanol) or not sedated (in those animals deemed too obtunded to give sedative) were scored. A subjective system, based on the amount of abnormal activity present, was used to score each EEG.

0 No abnormal activity was detected, normal.
1 Rare/occasional abnormal activity observed, mild.
2 Abnormal activity alternated with normal EEG, moderate.
3 Abnormal activity abundant, often obliterating the background EEG, severe.

In addition, details regarding the types of epileptiform discharges (spikes, sharp waves, slow waves, and/or spike waves) and their distribution (generalized, lateralized, over a particular brain region, and/or multifocal) of the events were noted. Responses in EEG activity associated with the administration of additional drugs or a change in the state were described.

### Related data

Status after treatment was recorded on most sea lions. When available, supporting diagnostic information (MRI and/or histology/necropsy) was compared to EEG findings on a given sea lion. These techniques have been described in previous publications (13, 30–32). In addition, those animals that were tagged and released were followed as part of another study (33). Others were evaluated behaviorally to better understand the impact of DA on memory and spatial ability (25).

## Results

### EEG findings

Some scores differed between readers but not by more than one value. In these instances, the higher of the two scores was used for that sea lion (refer to Supplementary Table).

#### Neurologically normal group

**Figure 2:**
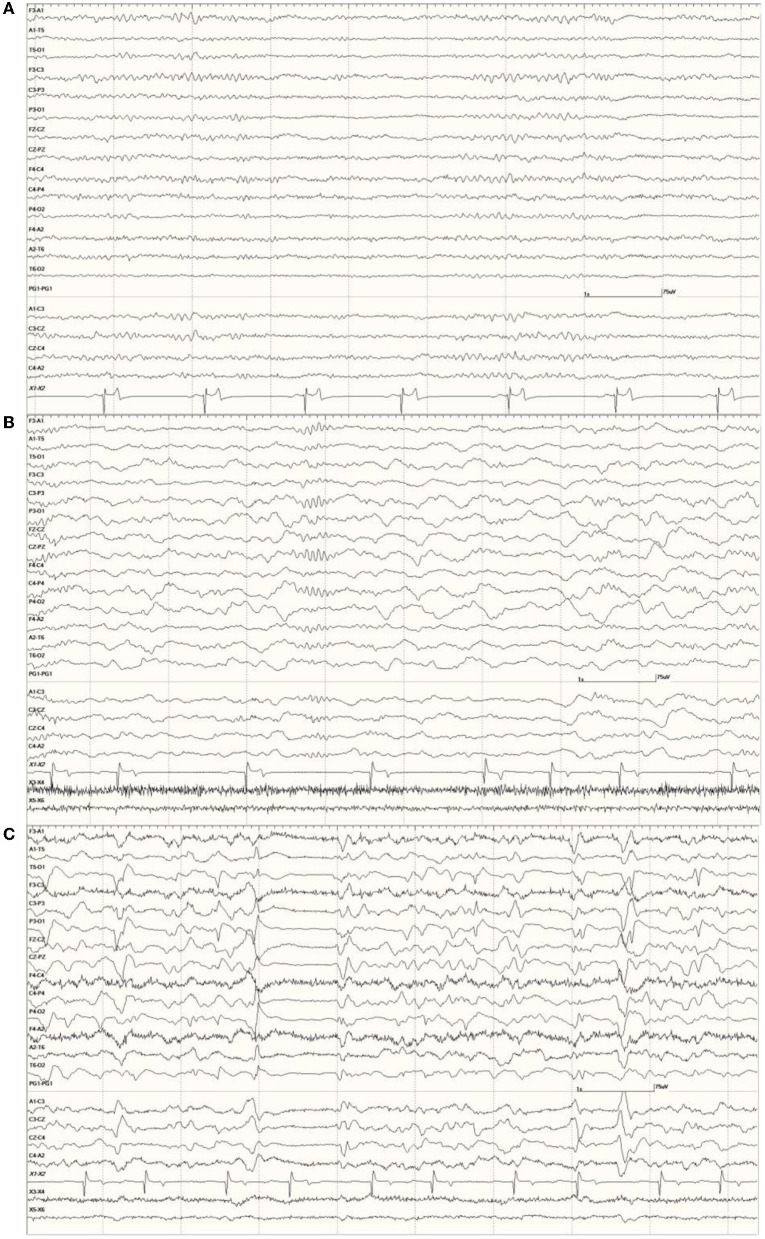
*Neurologically Normal* sea lion (7347) wakefulness EEG pattern with alternating beta activity (>13 Hz, low amplitude at middle and end) and alpha activity (8–13 Hz, medium amplitude at the beginning and between middle and end) **(A)**. Slow wave sleep in a *Neurologically Normal* sea lion (6326) with ongoing delta activity (<4 Hz high amplitude) and a well-developed sleep spindle before the 4 s mark (vertical line) with smaller ones at the beginning and end of this epoch **(B)**. Both had EEG scores of 0. The same sea lion (6326) during isoflurane anesthesia **(C)**. A 1-s suppression period occurred between the 3 and 4 s marks. Multiple sharp (duration 70–200 ms) and slow waves (>200 ms) can be seen. Superimposed muscle artifact (fast, darker, jagged-looking activity) is present bifrontally (F3 and F4).

Artifact was a common finding in these EEGs. It consisted primarily of muscle artifacts (especially in EOG channels), but it also included artifacts associated with eye, head, and generalized movement. Occasional myoclonic jerks were observed during EEG recordings and are described as follows: stereotyped twitches of the eyes, the nose, the head, or the body. Periods of wakefulness and slow-wave sleep (SWS) were recorded and consisted of:

*Wakefulness:* a mixture of low amplitude (<40 μV) beta activity (>13 Hz) often interspersed with higher (~60 μV) runs of rhythmic alpha activity (8–13 Hz), as shown in Figure 2A. This state was observed primarily only in the latter part of some EEGs (in those sea lions that did not receive general anesthesia and whose recordings were long enough for sedative effects to wear off or were given atipamezole and/or stimulation for reversal).

*Slow-Wave Sleep:* a background of ongoing large (maximum of 240 μV), random, slow (delta range, <4 Hz) waves and the intermittent appearance of sleep spindles (up to 80 μV, at 12–14 Hz with a duration of 0.5 s or less). This state was symmetrical in both hemispheres. An example is shown in Figure 2B. This state was observed throughout most or all of the EEGs in the majority of sea lions.

No rapid eye movement (REM) sleep was observed in any of these recordings.

In most individuals, the administration of medetomidine/dexmedetomidine was associated with long periods of SWS. The female *Neurologically Normal* sea lion (7158) was an exception, as the entire recording consisted of wakefulness. Other than a suspected increase in myoclonic activity, butorphanol did not appear to influence the EEG. Isoflurane had a profound effect on background activity, occasionally resulting in a burst suppression pattern (Figure 2C).

Scoring in the Neurologically Normal Group was defined, as follows:

0 = 5 sea lions (1 FP, 1 MP, 1 MY, and 2 MS).

1 = 9 sea lions (4 MP, 2 MY, and 3 MJ).

2 = 0 sea lions.

3 = 1 sea lion (MS).

**Figure 3:**
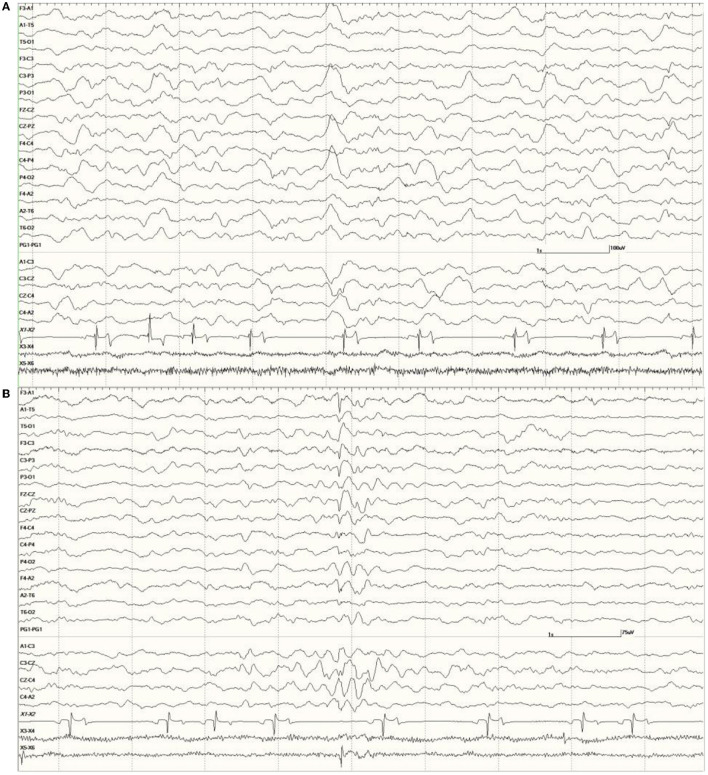
Recordings from *Neurologically Normal* sea lions with mild abnormalities (a score of 1). Sea lion 7155 recording with a large slow wave in the middle of the epoch **(A)**. An example of a typical generalized slow wave preceded by a deep positive (downward) deflection at the same time point in sea lion 8488 **(B)**.

**Figure 4:**
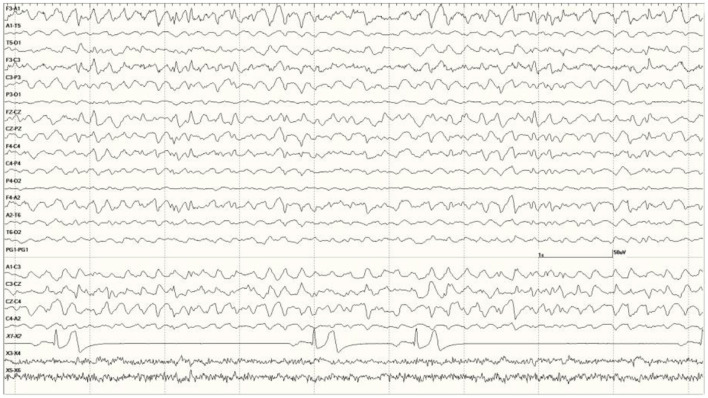
Segment of EEG from a sea lion initially thought to be *Neurologically Normal* (7689). Continuous generalized abnormal slow wave activity was present throughout this recording (EEG score = 3). The frequency varied between 4 and 5 Hz and may represent an electroencephalographic seizure (no clinical signs were apparent).

Occasional abnormal slow waves, some high in amplitude (400 μV), were a common finding in animals with an EEG score of 1 (Figure 3). In all cases, these slow waves differed from those of SWS in that they were uniform and tended to be generalized. Many were also preceded by a deep positive phase (Figure 3B). The single severely abnormal sea lion (a sub-adult male) in this group (7689), which presented for entanglement and was also a restrand, had continuous slow waves interspersed with sharp waves and spikes throughout the 21-min recording (Figure 4). This may represent an electroencephalographic seizure as no clinical sequelae were apparent in this individual.

#### Suspected DA toxicosis cases

Artifact was less frequent in this group of sea lions, as sedation effects tended to be more pronounced. Muscle artifact, when present, was primarily in the EOG channels. When the background was visible, it was primarily that of SWS for most of the recording.

**Figure 5:**
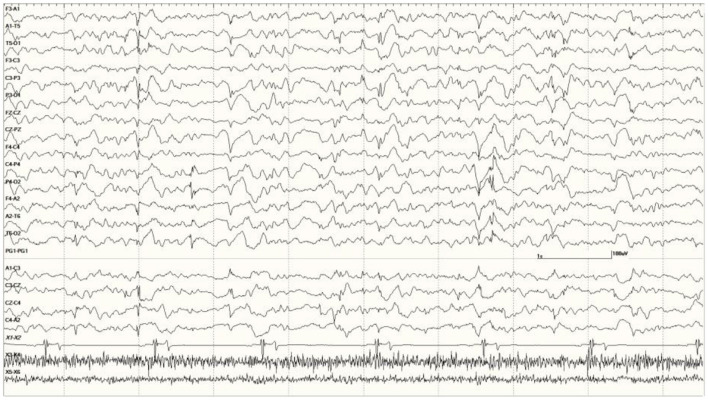
A sample of EEG from a *Suspected DA toxicosis* sea lion (8659, a score of 3) during slow-wave sleep. Several generalized large slow waves are apparent in addition to right hemisphere spikes (duration <70 ms, after the 1- and 2-s marks). A spike-wave discharge is shown between the 5- and 6-s marks. Muscle artifact is present in the EOG channels.

**Figure 6:**
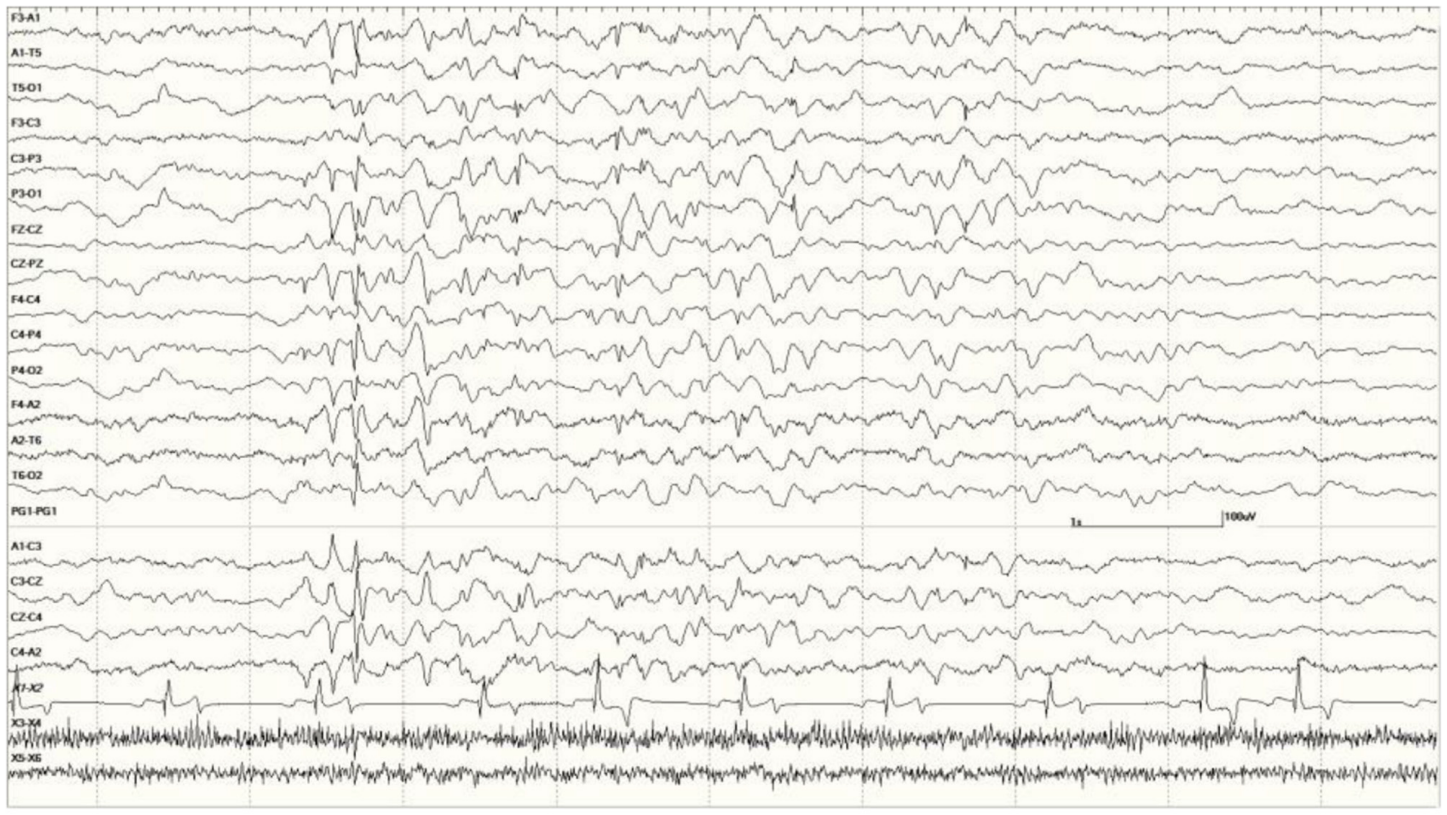
Five s burst of generalized abnormal activity consisting of slow waves, spikes, and sharp waves between normal slow-wave sleep background segments in *Suspected DA toxicosis* sea lion 6741 (a score of 3).

**Figure 7:**
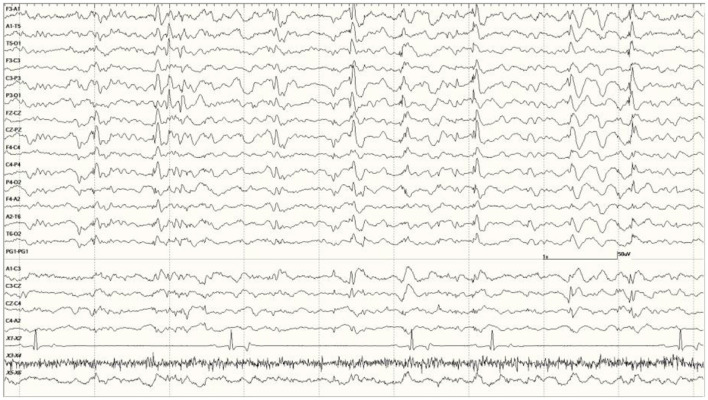
Quasiperiodic (irregular intervals) generalized and multifocal discharges in *Suspected DA toxicosis* sea lion 6887 (a score of 3).

**Figure 8:**
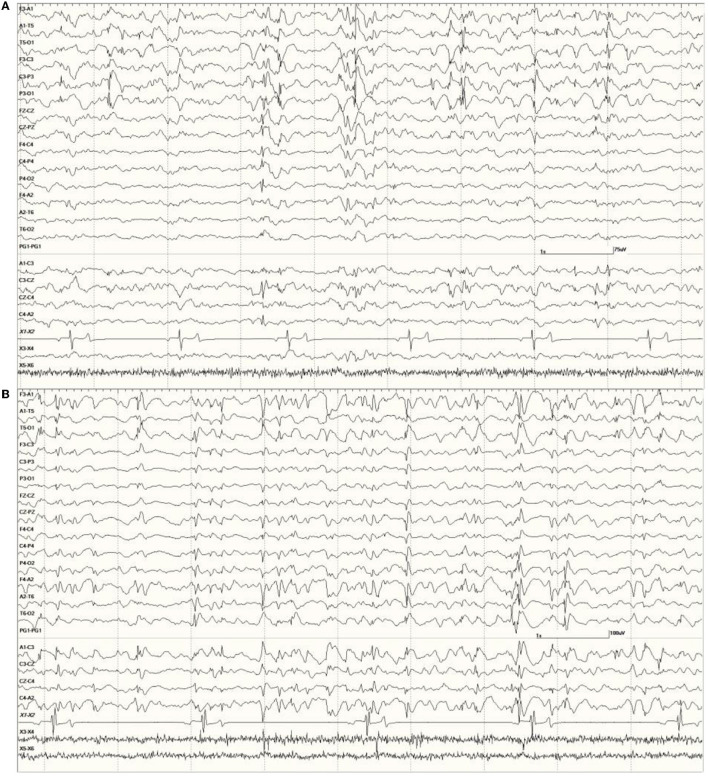
Primarily left-sided EEG abnormalities (spikes, sharp waves, and slow waves) with a few spike waves extending into the right hemisphere in *Suspected DA toxicosis* sea lion 6731 **(A)**. A segment of EEG in *Suspected DA toxicosis* sea lion 9935 demonstrating various abnormalities with a bitemporal distribution **(B)**. Both had a score of 3.

**Figure 9:**
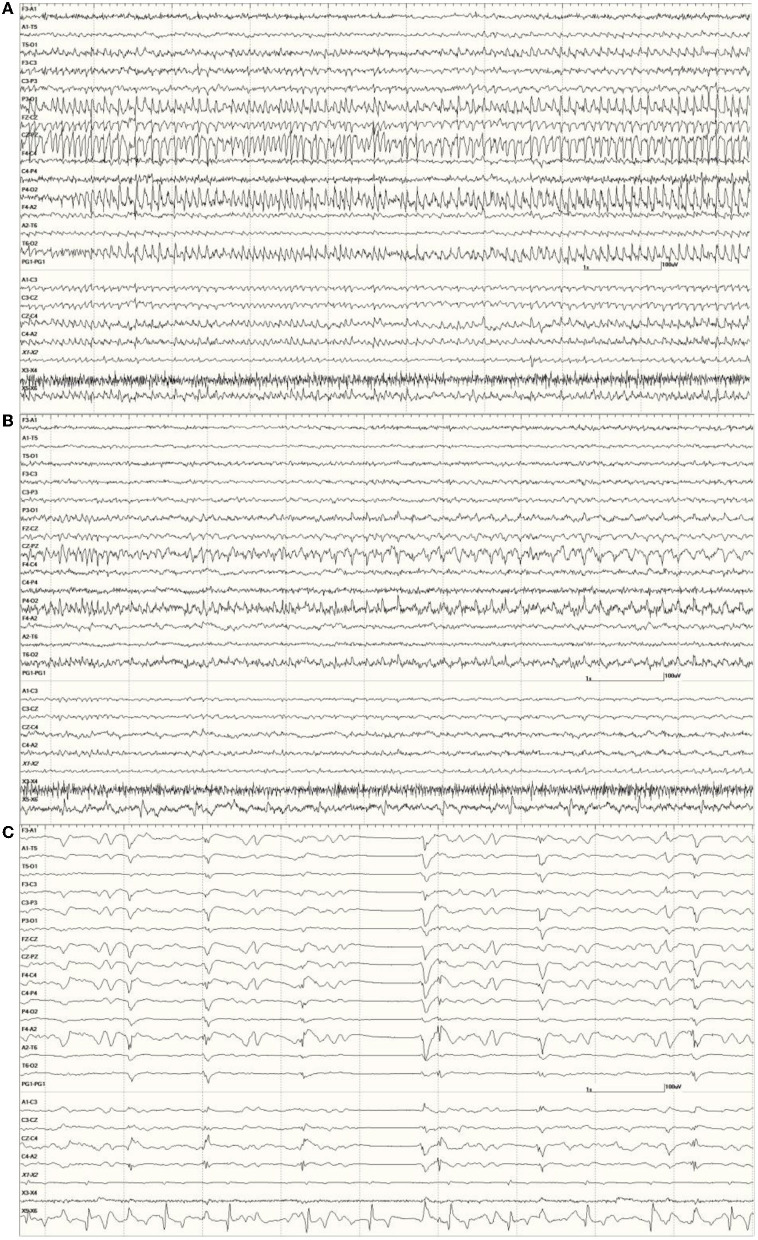
An unusual recording in *Suspected DA toxicosis* sea lion 7160 (a score of 3) consisting of continuous spikes **(A)**, spikes devolving into sharp waves **(B)**, both voltage maximum caudally (occipital region). This appears to be an electroencephalographic seizure. Isoflurane administration had profound effects on the EEG pattern **(C)**.

Mixed epileptiform discharges, consisting of

spikes (duration <70 ms),

sharp waves (70–200 ms),

slow waves (>200 ms), and

spike waves (a spike followed by a slow wave),

were common in the EEGs from this group. Some were single events (Figure 5), but others were bursts of generalized discharges sandwiched between periods of normal EEG (Figure 6). In the most severely affected sea lions, abnormal rhythmic activity was nearly continuous throughout the recording, similar to that seen in Figure 4. Occasionally, the discharges were irregularly periodic in occurrence (referred to quasiperiodic, Figure 7). The abnormal activity was most commonly generalized, but in some animals, epileptiform discharges were worse on one side (unihemispheric), only occasionally extending to the opposite side. Spikes were often multifocal (Figure 8A). All but one sea lion (7160) in this group displayed a mixture of these events. When multiple, slow-wave, and spike-wave bursts varied in frequency from 3 to 5 Hz and were often voltage maximal either frontally or occipitally, though in a few sea lions, it was bitemporal (Figure 8B). An unusual pattern was noted in sea lion 7160 that displayed nearly continuous spike/sharp wave activity for 25 min at which time isoflurane was administered (Figure 9). This recording is suggestive of an electrographic seizure.

**Figure 10:**
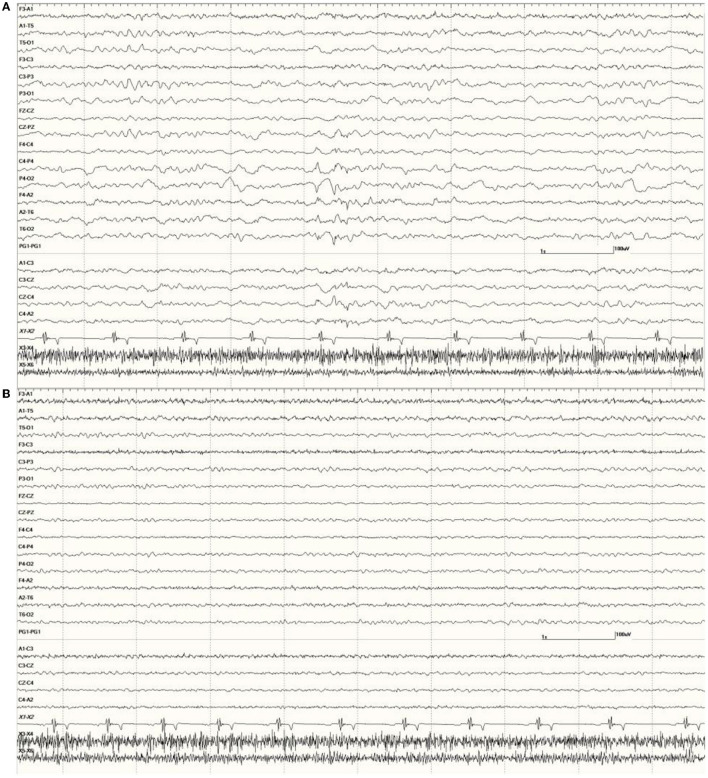
Change in abnormal activity associated with state. *Suspected DA toxicosis* sea lion 8659 (same as in Figure 5) near the end of the recording with a right-sided discharge during slow-wave sleep **(A)** and normal wakefulness **(B)**.

In several instances, waking the animal at the end of the recording, in some cases by administering atipamezole and/or stimulating (*via* nose or flipper taps), resulted in a dramatic improvement in the EEG (Figure 10, compared to Figure 5).

**Figure 11:**
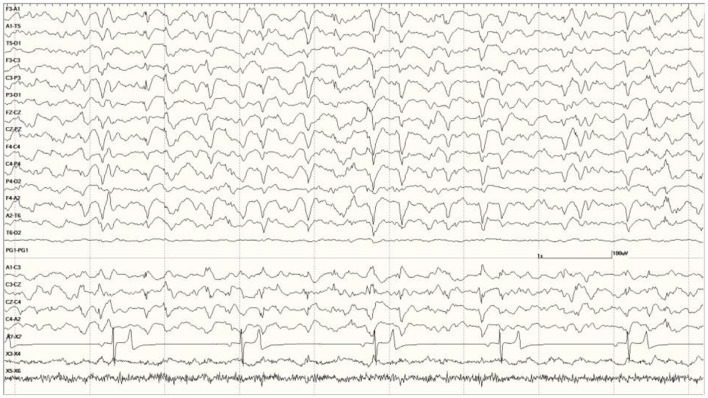
Change in EEG associated with isoflurane anesthesia in *Suspected DA toxicosis* sea lion 6731 (compare with Figure 8A).

As was mentioned for the *Neurologically Normal Group*, isoflurane was associated with dramatic changes in the EEG. Large slow waves, sharp waves, and spikes, similar in appearance to those seen with sedation during SWS, were present with this general anesthesia. These were always generalized, even in those sea lions that had focal or unihemispheric discharges prior to induction (Figure 11, compared to Figure 8A).

**Figure 12:**
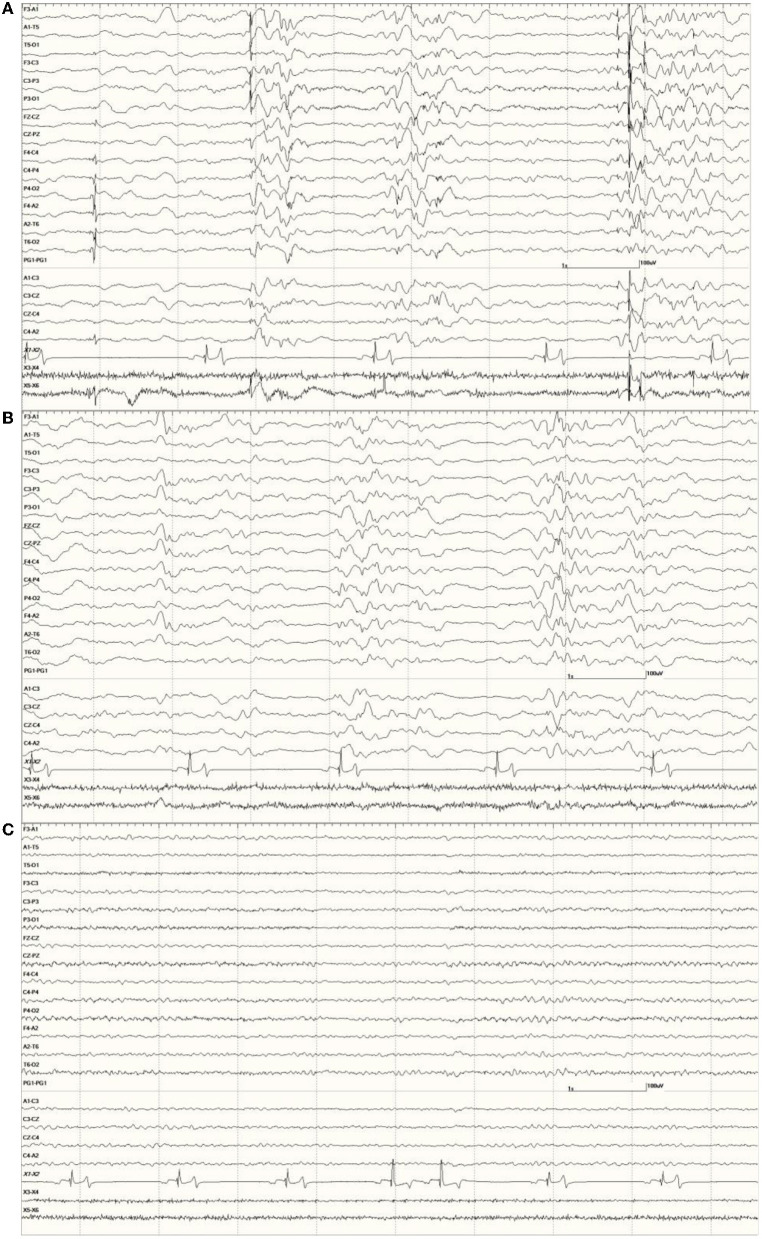
The effect of a benzodiazepine on the EEG in sea lion 6667 (a score of 2). Before administration, there are numerous spikes (only the largest one is likely blink artifact) **(A)**. Loss of abnormal fast activity after lorazepam administration **(B)** and disappearance of all abnormalities associated with wakefulness **(C)**.

The administration of benzodiazepines, midazolam, or lorazepam, during recordings, did not appear to reduce the presence of slow-wave discharges in the EEG but did reduce or eliminate spikes and sharp waves (Figure 12).

Scoring for this group of sea lions was as follows:

0 = 0 sea lions.

1 = 7 sea lions (1 MP, 1 MJ, 2 FS, and 3 FA).

2 = 23 sea lions (4 MY, 2 MJ, 1 MS, 2 FY, 2 FS, and 12 FA).

3 = 58 sea lions (5 MJ, 1 MS, 1 FP, 8 FY, 6 FS, 30 FA, 6 F age unknown, 1 gender and age unknown).

Those sea lions that had repeat recordings after receiving treatment with phenobarbital for 7 to 28 days showed minimal changes in EEG scores, at most, a one-step improvement (e.g., from 3 to 2).

### Related data

#### Neurologically normal group

Only two sea lions in this group (8052 and 7689) had MRI studies. The former was read out as normal but no results were reported on the latter. None had necropsy/histological studies. Seven of these sea lions were released after treatment, two died (6326 and 9483), and five were placed at other facilities with 7689 being no status given.

#### Suspected DA toxicosis cases

**Figure 13:**
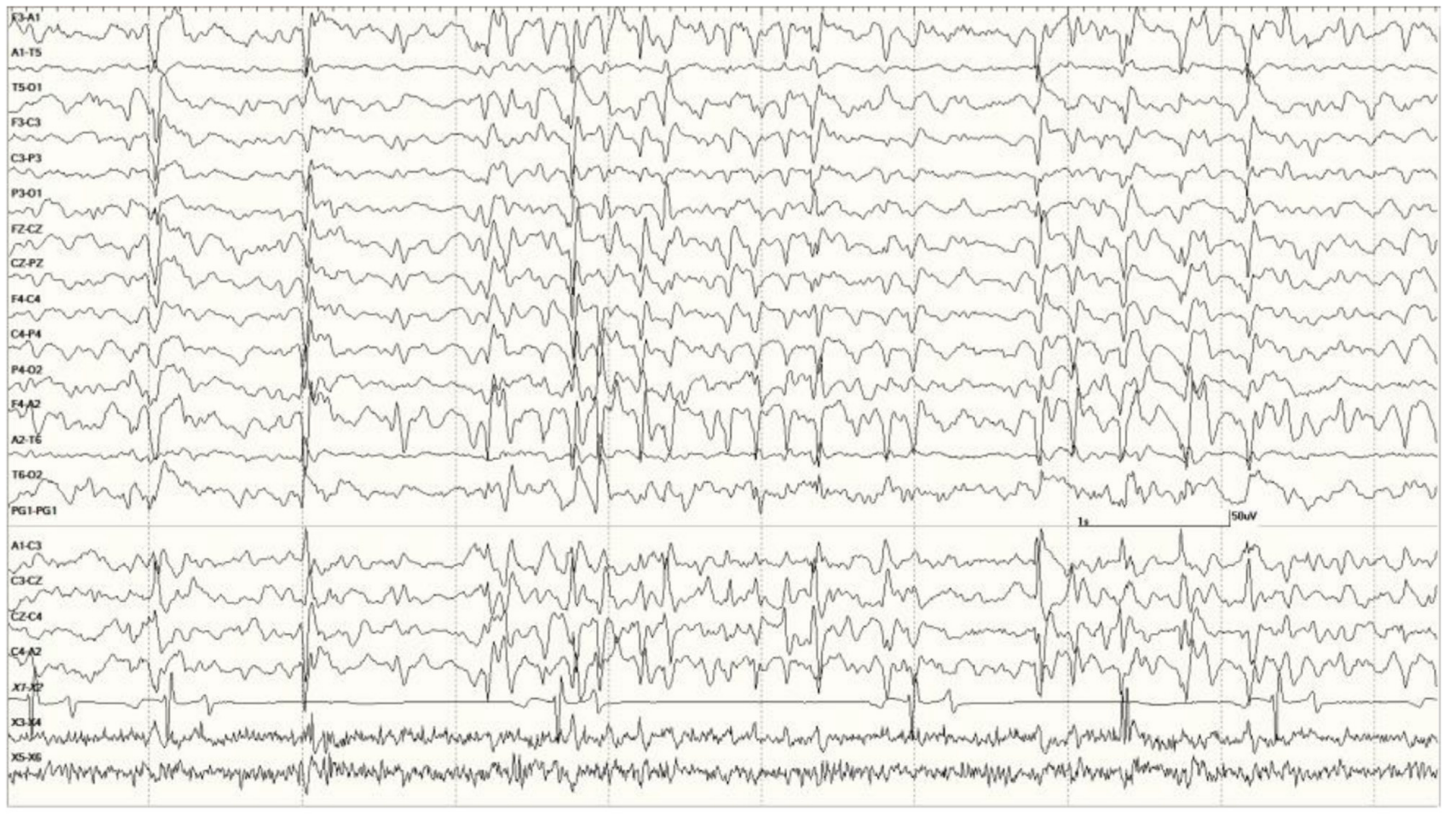
Individual generalized events and bursts of sharp and slow waves in *Suspected DA toxicosis* sea lion 9724 (a score of 3). This animal was diagnosed with white matter lesions on MRI but hippocampi were considered normal. Mild encephalitis and cardiomyopathy were found at necropsy consistent with a protozoal etiology.

In this group, 22 sea lions received MRIs. Findings in one (9724) stated that the hippocampi were normal but white matter lesions were present (Figure 13). The EEG score in this animal was 3. Most (*N* = 12) had unilateral hippocampal (HC) necrosis, with five on the left and seven on the right. Ratings varied on the left with three having mild atrophy (6710, 6859, and 9690), one with severe (6508), and one was not rated (7745). For those with right-sided atrophy, two were moderate (6405 and 6433), three had severe (4589, 6510, and 6707), and two were not rated (8722 and 8883). Six had bilateral HC atrophy with 6887 showing more severe changes on the right than the left. The other cases consisted of two with mild (6667 and 6904) and three with severe bilateral HC atrophy (6740, 6804, and 9821). One each with severe left HC atrophy (6508), severe right-sided atrophy (6510), and severe bilateral HC atrophy (6804) had an EEG score of 1. Another two sea lions (9255 and 9931) in this group had MRI but no results were available.

**Figure 14:**
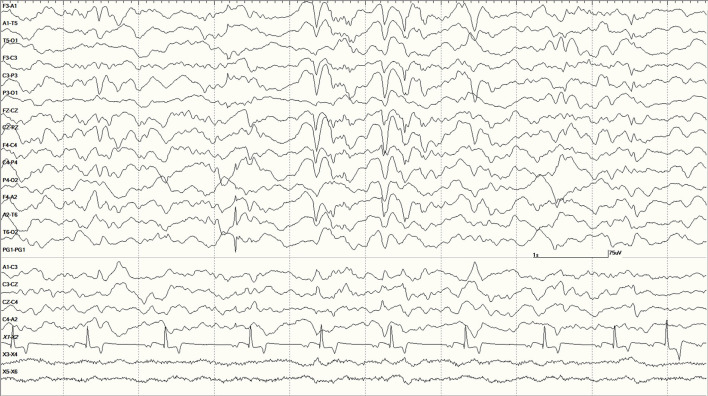
Large slow waves and a positive (pointing away) right temporal spike between the 3 and 4 s marks in *Suspected DA toxicosis* sea lion 6079 (a score of 1). This animal was diagnosed with porencephaly at necropsy.

**Figure 15:**
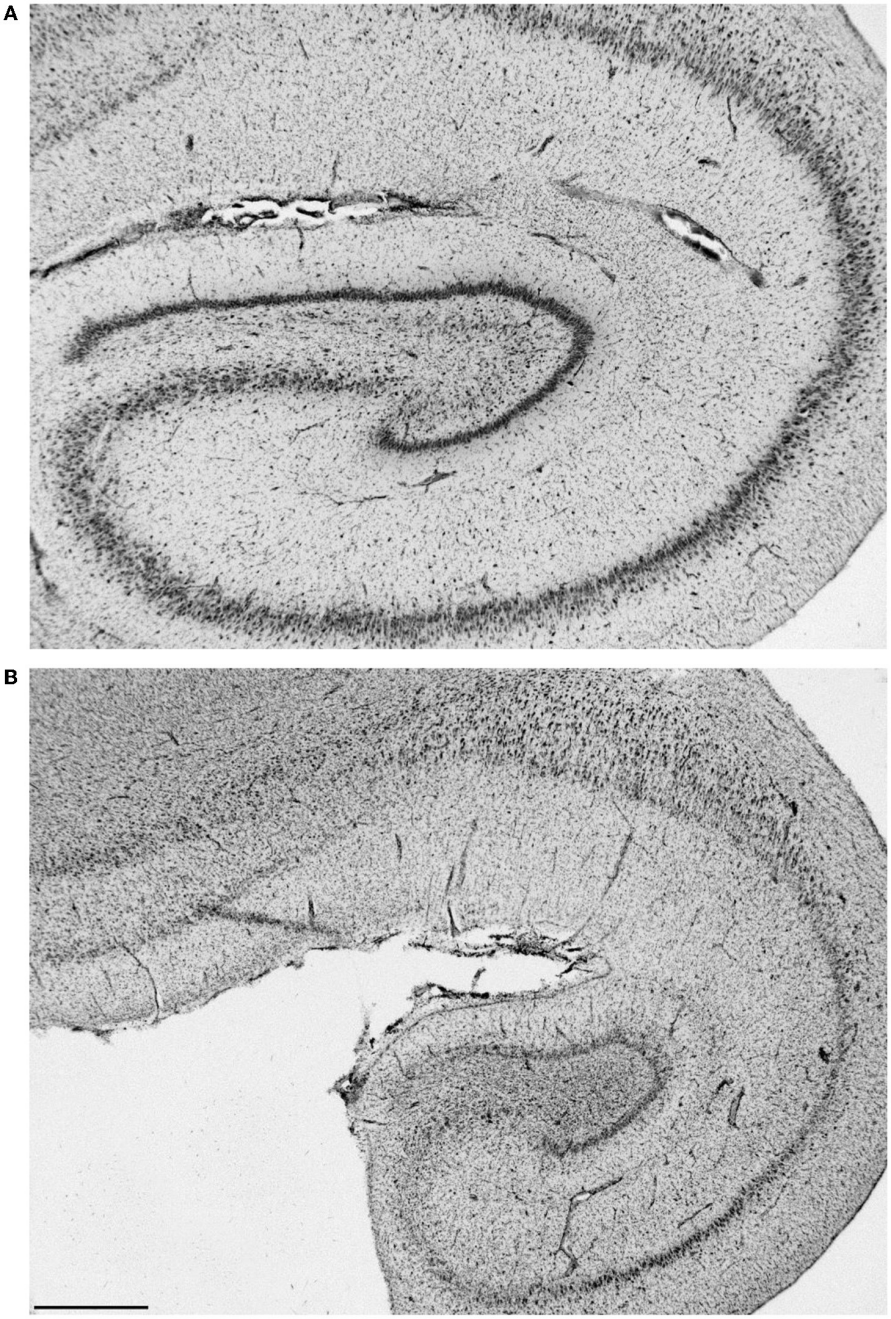
Histological section of a normal left hippocampus from sea lion 9724 **(A)**. Hippocampal section from one (9821) with severe bilateral atrophy [**(B)**, left shown]. Bar is 1 mm, the same scale for both.

Brain (primarily hippocampal) necropsy/histology results were provided on 24 sea lions in this group. Various findings are as follows: two no gross HC lesions (9925 and 9938), two perivascular edema (6731 and 8883), one brain necrosis (6365), and one porencephaly (6079; Figure 14). Sea lion 6731 also had signs of a brain hemorrhage. Five had left-sided HC atrophy (two mild [6489 and 6859], one moderate [6318], and two severe [6508 and 6804]) with seven diagnosed with right-sided atrophy (two moderate [6090 and 6405] and five severe [4589, 6510, 6676, 6707, and 6887]). There were two cases of moderate bilateral atrophy (6123 and 6741), one with severe left-sided atrophy and moderate right-sided atrophy (6804) and two with moderate unilateral HC loss (side not mentioned; 6084 and 6290). One case (9574) was described as having diffuse atrophy. One of the sea lions with an incomplete number (805?) had severe left HC atrophy and another (8722) was described with severe loss. A normal left hippocampus (9724) and a severely atrophied left hippocampus (9821) are shown in Figure 15. One adult female without HC changes (9938) was found to have carcinoma throughout the abdominal cavity with enlarged lymph nodes, abnormal kidneys, and multiple liver nodules. Despite an EEG score of 3, this sea lion's brain was grossly normal. On necropsy, sea lion 9724 had mild encephalitis, vaginal carcinoma, myocarditis, and verminous (lungworm) pneumonia. Brain and heart changes were consistent with a protozoal etiology. No evidence of metastatic disease was present, so the carcinoma was likely early stage. On radiography, lead fragments were found in one sea lion (6741) and a 9-mm bullet in another (6804). In both cases, they were lodged below the skin of the neck and appeared to be isolated by fibrous tissue.

For the *suspected DA toxicosis* sea lions, 13 were released, 51 were euthanized, 15 died, and 10 had no status listed. In contrast to the *Neurologically Normal Group*, none were placed. Eight of the released animals were fitted with satellite tags to study their movement following release. One (6904) stayed in Monterey Bay but restranded with seizures and was euthanized. Another (6887) was found to be disoriented on a police car in San Francisco and was also euthanized. Two others in this subset were euthanized with the remaining four animals' statuses unknown.

A summary of all sea lion data included in this study is shown in the Supplementary Table.

## Discussion

Recording EEG in free-ranging, stranded California sea lions was possible with the use of a_2_ agonists in most cases. As has been reported in cats, dogs, and horses, these drugs produce SWS that has the same characteristics (background and transient events) as that which occurs naturally (34–36). This state has been associated with a greater probability of detecting abnormal activity in an EEG recorded from a patient with epilepsy (37, 38). It holds true across species (39), and sea lions are no exception. Dramatic improvement in EEG recordings (decrease/disappearance of epileptiform activity) was seen following transitions from sleep to wakefulness in all sea lions where state changes occurred. Similar to the findings in horses, isoflurane had confounding effects on the EEG, even in animals determined to be neurologically normal (Figure 2C) (40).

EEG recordings were sensitive, picking up abnormalities in most sea lions during SWS (even those that were not neurologically impaired) but not specific, as similar features were observed in animals diagnosed with porencephaly, cancer, metabolic disease, or white matter lesions. In human medicine, EEG patterns are not pathognomonic for specific disorders. Similar patterns can be associated with a variety of etiologies, cerebral or systemic, such as, cerebral hypoxia, diffuse encephalitis, sepsis, severe metabolic disease, non-convulsive status epilepticus, drug overdose/withdrawal, or hypothermia (41). PDs have been reported with these maladies and were noted in the EEG from the elderly human patient with amnesic shellfish poisoning (18). None of these sea lion EEGs met the PD criteria (refer to Knipe, et al. in this issue), though sea lions 6090, 6667, and 6887 (Figure 7) had occasional quasiperiodic discharges in their EEG recordings.

Multiple studies of the neurological effects of DA have been performed in rats. Some involved DA injections (either into the peritoneum [IP] or directly into the hippocampus) of sublethal doses of the toxin and determination of the time to onset of stereotypic behaviors, such as scratching, lethargy, shaking, tremor, or seizure (42–44). A few also describe the associated electrocorticography (ECoG) findings (43, 44). Latencies to onset of interictal spikes decreased as the dose for those rats hippocampally injected increased with a concurrent increase in the frequency of spikes (43). Clinical seizures were apparent at the 3 highest doses only (100, 200, and 300 pmol). Differences in the display of data make comparisons with the Scallet report difficult, as their analyses were done using fast Fourier transform (FFT) so raw EEG details are lacking (44). They describe significant increases in delta and theta power (voltage squared, in the 1.25–4.50 Hz and 4.75–6.75 Hz frequency bins, respectively) primarily at the higher dose (4.4 mg/kg IP). Theta power in the low-dose (2.2 mg/kg) group only achieved significance 95 min post-injection compared to 30 min for the high-dose group, delta power never did achieve significance in the low-dose group (but did at 35 min for the high-dose group). Higher frequency bands, alpha 1 (7.00–7.50 Hz), alpha 2 (9.75–12.50 Hz), and beta (12.75–18.50 Hz), took longer in both groups to attain significance (75–85 min for high, 110 min for low). Spikes are mentioned but cannot be seen in the figure, as the time base is very compressed (60 s/division instead of the usual 1 s/division).

Early cases of DA toxicosis in sea lions were associated with mass strandings that coincided with algal blooms of *Pseudo-nitzschia australis* (45). This DA exposure was thought to induce damage initially that may later go clinically undetected for some time (a latent period), only to show up eventually as epilepsy (46). This was certainly the case with the human patient described by Cendes who developed complex partial epilepsy 1 year after ingestion of mussels containing DA despite being seizure free for the previous 7.5 m (18). Age susceptibility may have played a role in amnesic shellfish poisoning. Of the 107 confirmed human cases, 49 (46%) were between the ages of 40 and 59 years and 38 (36%) were 60 years or older, with one patient's age unknown (17). An MRI study of 53 sea lion DA toxicosis cases found more severe losses in hippocampal and parahippocampal gyrus volumes in adult animals (47). The authors attributed this to a combination of older animals potentially being more susceptible to the toxin and accumulated damage being induced by the recurrent seizures themselves. This is in contrast to Simeone's report that the developing brain of the fetus/neonate is less likely to be protected from DA effects by virtue of its incompletely formed blood–brain barrier (48). In numerous instances, sea lions exposed to DA *in utero* do not make it to term (22, 23) and those that do risk further DA exposure by way of the dam's milk (26). The *Neurologically Normal Group* consisted of 40.0% pups (six out of 15) but only two (7158 and 8031) had normal EEGs (a score of 0) and four were 1s. The female pup's (7158) EEG recording did not contain any periods of sleep; therefore, the lack of abnormalities may be state-related. All of these pups could have been exposed to DA with the potential to develop neurological signs over time (even without additional exposure). This age group represented only 2.3% (two of 88) of the *Suspected DA Toxicosis Group* with one receiving a score of 1 and the other with a score of 3. The former was hypoglycemic when admitted, which likely contributed to this sea lion's neurological status. Drawing conclusions about the effect of age requires further study.

Gender appears to be an important factor in this malady, as 81.8% (72 of 88) of the sea lions in the *Suspected DA Toxicosis Group* are females, compared to 6.7% (one in 15) in the *Neurologically Normal Group*. This finding has been attributed to the species' foraging behavior with only females feeding close to rookeries year round where they are more likely to encounter the toxin (46).

Domoic acid preferentially binds to the kainic acid subtype of glutamate receptors prevalent in the CA3 region of the hippocampus (29). It binds both presynaptically and postsynaptically where it induces elevations in intracellular calcium levels, leading to excitotoxic damage such as neuronal loss and astrocytosis. Memory and spatial ability are critical functions of the hippocampus, which can be severely impacted by DA toxicosis/amnesic shellfish poisoning. This brain structure is also involved in the development of some forms of epilepsy. Cats (49), humans (50), and sea lions (29, 32, 46) all have this in common. White matter pathology in the fornix and increases in hippocampal-thalamic connectivity are reportedly similar in both DA toxicosis sea lions and humans with mesial temporal lobe epilepsy (51). In addition to being a sentinel species that can provide valuable information regarding the presence of toxins in the environment, the sea lion may also be an animal model for the most common form of epilepsy in humans. Future sea lion EEG studies can be beneficial in determining the functional aspects of this disorder in conjunction with those involving imaging and pathological findings.

Despite the limitations of this study (no known history prior to stranding [with the exception of restrands]), a lack of truly normal controls (sea lions with no known history of DA exposure or other medical conditions), various missing data points, and minimal follow-up [consisting of telemetry data on the locations of released animals with tags and the status of sea lions placed in other facilities], some valuable knowledge has been gained.

## Conclusion

High-quality recordings of normal EEG and a range of abnormal EEG, representing a few different etiologies, were obtained from this population of formerly free-ranging sea lions (those living under natural conditions up until the time they stranded and were treated at a rehabilitation facility). These data can be applied to future studies in this species but can also be used to compare EEG findings across species.

## Data availability statement

The datasets presented in this article are not readily available because EEGs are stored on proprietary software. Requests to access the datasets should be directed to DW, colette.neurophys@gmail.com.

## Ethics statement

Ethical review and approval was not required for the animal study because the work was authorized under the US Marine Mammal Protection Act by Scientific Research permit no. 932-1489-00.

## Author contributions

DW, MH, SD, FG, KL, and BT designed the study. LW, TG, FN, BB, PB, and VH were involved in data acquisition/analysis. DW wrote the manuscript with editing provided by SD, KL, PB, FG, and VH. All authors contributed to the article and approved the submitted version.

## Footnotes

^1^Due to differences in maturation, only pup and yearling designations are the same for both sexes. There are no juvenile females, so subadult and adult ages differ between males and females. No adult males (greater than 8 years of age) were present in this study, whereas adult females were the most represented group at 43.7% (45 out of 103).
